# Single-port versus multi-port laparoscopic left lateral sectionectomy using articulating instruments: a comparative study

**DOI:** 10.1007/s00423-026-04018-1

**Published:** 2026-04-01

**Authors:** Rak-Kyun Oh, Seok-Hwan Kim

**Affiliations:** 1 Division of Acute Care Surgery, Department of Surgery, Chungnam National University Hospital, Daejeon, Republic of Korea; 2 Department of Surgery, College of Medicine, Chungnam National University, Daejeon, Republic of Korea; 3 Division of Hepatobiliary and Pancreas Surgery, Department of Surgery, Chungnam National University Hospital, 282 Munhwa-ro, Jung-gu, Daejeon, 35015 Republic of Korea

**Keywords:** Single-port laparoscopy, Laparoscopic liver resection, Left lateral sectionectomy, Articulating instruments, Minimally invasive surgery, Hepatectomy

## Abstract

**Background:**

Single-port laparoscopic surgery (SPLS) for left lateral sectionectomy (LLS) offers potential advantages in reducing invasiveness and improving cosmesis, but its adoption remains limited because of technical challenges, including loss of triangulation and instrument collision. We hypothesized that using articulating instruments could overcome these limitations and allow SPLS to achieve outcomes equivalent to conventional multi-port laparoscopic surgery (MPLS).

**Methods:**

A retrospective cohort of 77 patients undergoing LLS was analyzed (SPLS, *n* = 42; MPLS, *n* = 35). SPLS was performed via a single umbilical incision using articulating instruments. Perioperative outcomes were compared.

**Results:**

Baseline demographics and tumor characteristics were similar. All procedures were completed laparoscopically without conversion to open surgery or additional port placement. No significant differences were found in operative time, estimated blood loss, R0 resection rate (100% in both), or postoperative complication rates (SPLS 2.4% vs. MPLS 5.7%). No patient in the SPLS group required a blood transfusion, compared to two patients (5.7%) in the MPLS group (*p* > 0.05). The SPLS group had a significantly shorter hospital stay (5 vs. 6 days, *p* = 0.02). No mortality, reoperation, or port-site complication occurred.

**Conclusions:**

In selected patients and in the hands of experienced surgeons, SPLS using articulating instruments is a safe and effective alternative to conventional multi-port laparoscopy, achieving comparable perioperative and oncologic outcomes with a shorter postoperative hospital stay. These findings support articulating instrument-assisted SPLS as a feasible and high-quality approach for minor liver resection in experienced centers.

## Introduction

Laparoscopic left lateral sectionectomy (LLS) has become a standard minimally invasive liver procedure, offering well-established advantages over open surgery, including reduced postoperative pain, shorter hospital stays, and superior cosmetic outcomes [1–3]. To further minimize surgical trauma and enhance cosmesis, single-port laparoscopic surgery (SPLS) has been introduced, allowing the entire operation to be performed through a single umbilical incision [4–6]. Despite these potential benefits, the adoption of SPLS in liver surgery has been cautious owing to considerable technical challenges—particularly instrument crowding and loss of triangulation—which contribute a steep learning curve [7–9]. Articulating laparoscopic instruments have been introduced to overcome the inherent ergonomic limitations of single-port surgery by restoring internal triangulation and reducing instrument collision within the confined operative field.

Given its favorable anterior and peripheral location, LLS is considered the most suitable hepatic procedure for implementing SPLS. Since the first report by Aldrighetti et al. in 2010, several studies have demonstrated its feasibility in selected patients [10]. A randomized controlled trial comparing SPLS with conventional multi-port LLS (MPLS) for benign lesions reported comparable operative outcomes but a shorter hospital stay in the SPLS group [3]. Furthermore, a meta-analysis confirmed that SPLS is a safe and effective alternative to MPLS, although high-quality evidence remains limited.

**Fig. 1:**
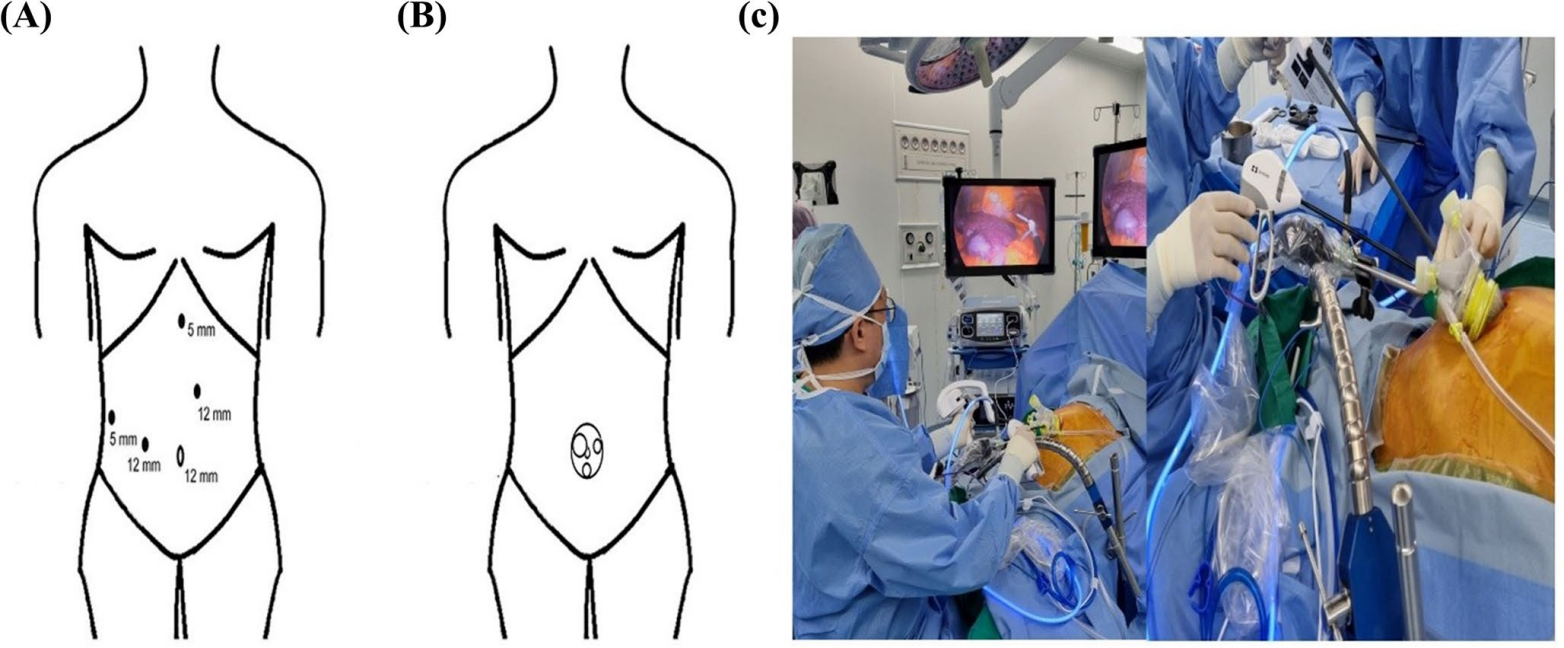
Comparison of Operative Setups for Laparoscopic Left Lateral Sectionectomy. (**A**) Standard multi-port laparoscopic surgery (MPLS) setup, demonstrating the typical placement of four trocars for camera and working instruments. (**B**) Single-port laparoscopic surgery (SPLS) setup, with a single multi-channel port placed within a 3-cm umbilical incision. (**C**) Intraoperative photograph showing the surgeon's ergonomic posture while performing SPLS with articulating instruments held in a pistol-grip fashion, minimizing external instrument clashing

**Fig. 2:**
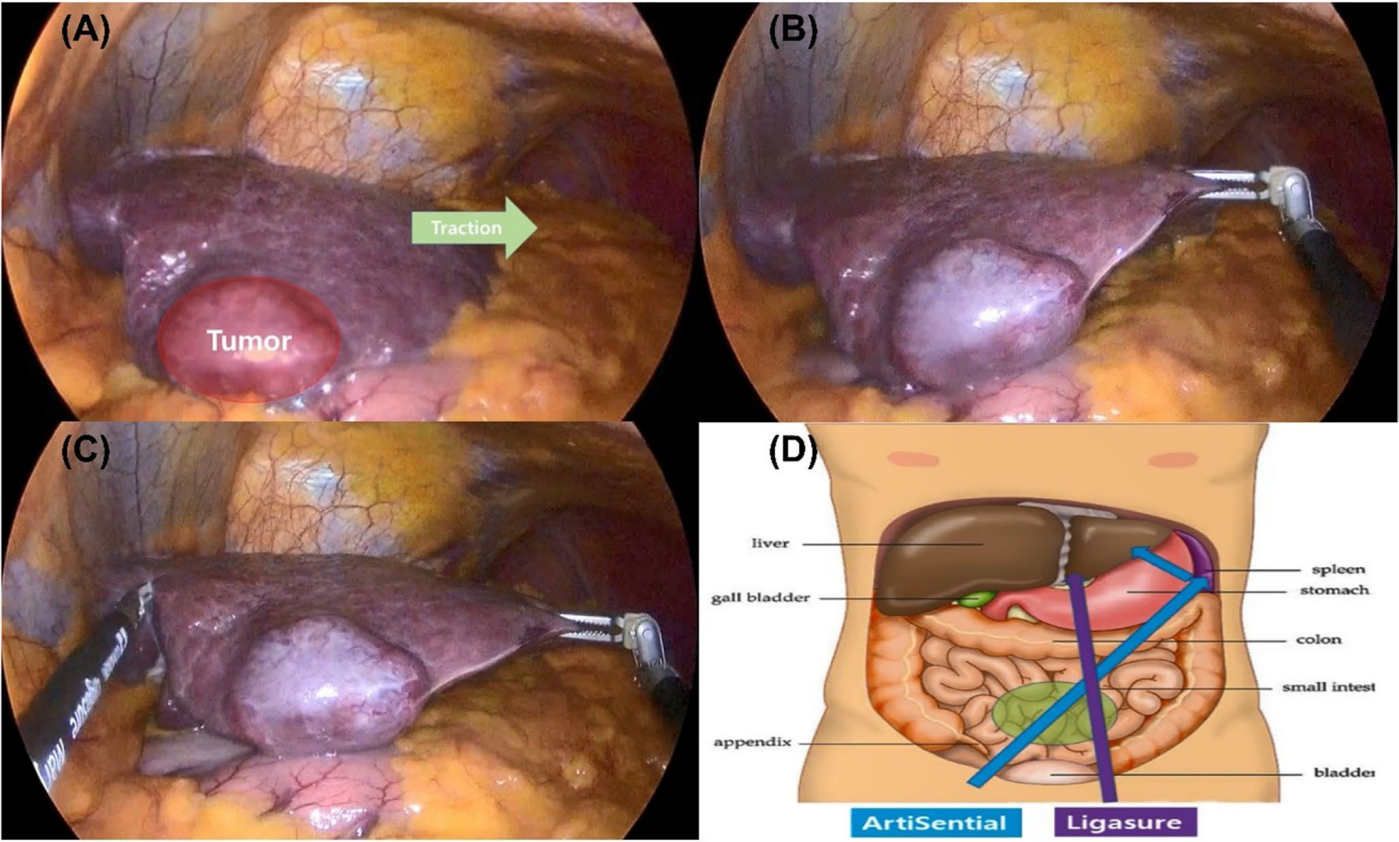
Intraoperative View Demonstrating the Advantage of Articulating Instruments in SPLS. (**A**) Initial intraoperative view showing limited exposure and ineffective counter-traction when straight instruments are constrained by parallel alignment in a single-port approach. (**B**) An articulating grasper provides effective traction despite crossed instrument entry. (**C**) Stable parenchymal manipulation during liver transection achieved without instrument collision (“sword fighting”). (**D**) Schematic illustration showing how articulating instruments restore instrument alignment and triangulation using articulating instrument in SPLS

To overcome the ergonomic limitations inherent to SPLS, newly developed articulating laparoscopic instruments provide wrist-like freedom of motion, restoring triangulation and enhancing dexterity within confined operative spaces (Fig. 2). In our institution, conventional rigid instruments were used only for MPLS, as preliminary experience with non-articulating SPLS revealed substantial ergonomic constraints and reduced instrument maneuverability. The difference in port placement and operative setup between MPLS and SPLS is illustrated in Fig. 1.

## Materials and methods

### Study design and patients

**Fig. 3:**
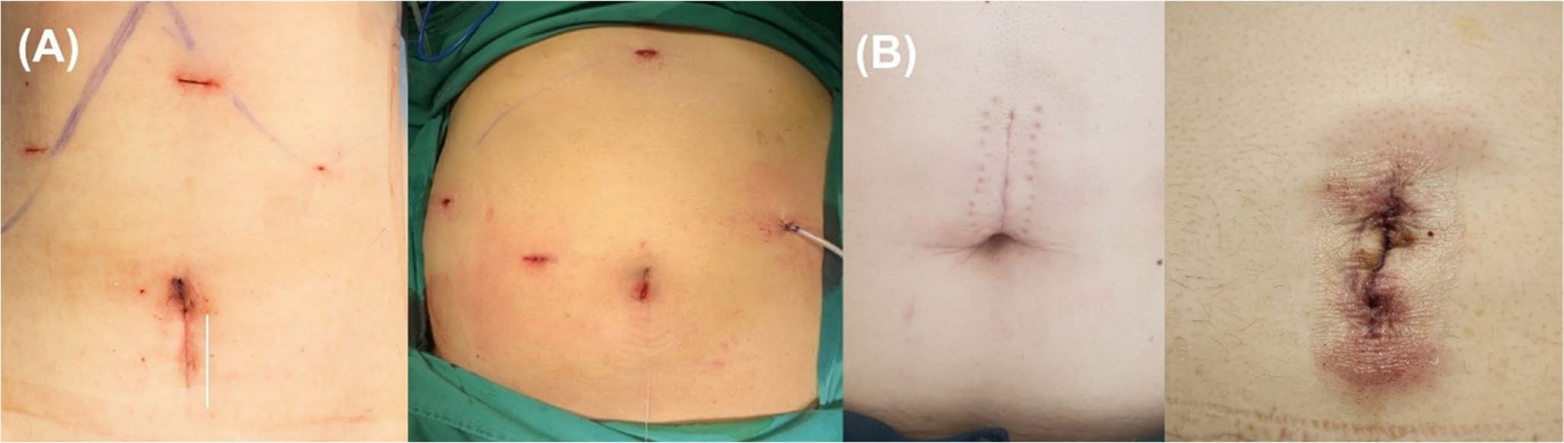
Postoperative Cosmetic Appearance. (**A**) Typical scarring after a conventional multi-port LLS, showing multiple small incisions in the upper abdomen. (**B**) The postoperative cosmetic appearance after single port LLS. The single incision is hidden within the umbilicus, resulting in a minimal scar. Improved cosmesis is a frequently cited potential benefit of the single-port technique

We performed a retrospective review of consecutive patients who underwent laparoscopic LLS at our institution from March 2018 to December 2024. Patients were categorized into SPLS or MPLS groups based on the surgical approach. During the study period, SPLS was offered as a preferred option for candidates deemed suitable for single-port access based on body habitus and preoperative imaging. All SPLS and MPLS procedures were performed by the same hepatobiliary surgeon. Cases requiring conversion to open surgery or those combined with additional procedures were excluded from the analysis (Figs. 2 and 3).

### Preoperative assessment

All patients underwent a standardized preoperative evaluation including contrast-enhanced CT or MRI, liver function tests, coagulation profile, viral hepatitis serology, and routine cardiopulmonary evaluation. Tumor characteristics—such as number, maximum diameter, and vascular involvement—were reviewed in a multidisciplinary hepatobiliary meeting to determine resectability. For malignant lesions, clinical staging was assigned using established radiologic criteria, and pathological grade was recorded postoperatively. History of prior abdominal surgery was reviewed for all patients, and no case exhibited adhesions severe enough to preclude laparoscopic access or alter port placement. All patients met institutional safety requirements for minimally invasive liver resection and were classified as Child-Pugh class A.

### Surgical technique

#### Single-Port Laparoscopic LLS (SPLS)

The SPLS procedure was performed through a 2.5–3 cm umbilical incision using a multi-channel single-port device, as shown in Fig. 1B. To overcome the ergonomic limitations inherent to SPLS, articulating laparoscopic instruments (ArtiSential^®^, LivsMed, Korea) were routinely employed to restore internal triangulation and improve instrument maneuverability within the confined operative field. Parenchymal transection was performed using the conventional clamp-crushing technique, supplemented by an articulating energy device and bipolar forceps. The left hepatic vein was divided with an articulating endoscopic stapler. The resected specimen was retrieved in a protective bag through the umbilical incision, which rarely required extension. No additional trocars were placed in any SPLS cases.

#### Multi-Port Laparoscopic LLS (MPLS)

A standard MPLS was performed using three or four trocars in a conventional figuration, as shown in Fig. 1A. The operative principles were identical to those used in the SPLS approach, except that conventional rigid laparoscopic instruments were used. Intraoperative ultrasound was employed as needed, and a surgical drain was placed at the surgeon’s discretion. All patients were managed according to a standardized institutional enhanced recovery after surgery (ERAS) protocol.

### Postoperative care and discharge criteria

Postoperative management followed the institutional ERAS protocol, emphasizing early ambulation, prompt removal of the urinary catheter, and structured advancement of oral intake. Patients were considered eligible for discharge when all of the following criteria were met: (1) adequate pain control with oral analgesics, (2) tolerance of a soft or solid diet without nausea, (3) independent ambulation, and (4) absence of fever or other signs of significant complications. If a surgical drain was placed, it was typically removed once the output was serous and less than 100 mL/day.

### Outcome measures and definitions

Data were collected on patient demographics, tumor pathology, and perioperative outcomes. Operative time was defined as the duration from skin incision to wound closure. Tumor size was defined as the maximum diameter of the dominant lesion in patients with multiple tumors. Postoperative complications were evaluated according to the Clavien-Dindo classification, with grade ≥ III considered clinically significant. The primary outcomes included operative time, estimated blood loss, need for transfusion, R0 resection rate, postoperative complication, and length of hospital stay.

### Statistical analysis

Continuous variables were compared using the Student’s t-test or Mann-Whitney U-test, and categorical variables were analyzed using the chi-square or Fisher’s exact test, as appropriate. A two-tailed p-value < 0.05 was considered statistically significant. No formal power calculation was performed, as this was an exploratory study. Statistical analyses were conducted using SPSS version 26 (IBM Corp, Armonk, NY, USA).

### Ethical approval

The study was conducted in accordance with the ethical principles of the Declaration of Helsinki and was approved by the Institutional Review Board of Chungnam National University Hospital (IRB No. 2022-04-036-006). The requirement for informed consent was waived due to the retrospective study design. The study adhered to the Strengthening the Reporting of Observational Studies in Epidemiology (STROBE) guidelines.

## Results

### Patient and tumor characteristics

A total of 77 patients undergoing laparoscopic LLS were included in the analysis: 42 in the SPLS group and 35 in the MPLS group. As summarized in Table 1, the two groups were well balanced in terms of baseline demographics and clinicopathological characteristics. The median age was 63 years in the SPLS group and 65 years in the MPLS group (*p* = 0.724), sex distribution (male: 57.1% vs. 54.3%, *p* = 0.652) and median body mass index (BMI) (25.6 vs. 27.2 kg/m², *p* = 0.348) were comparable between the groups. All patients were classified as Child-Pugh class A. The prevalence of liver cirrhosis was similar (33.3% vs. 34.2%, *p* = 0.732).

**Table 1:** Baseline demographic, liver background, and tumor characteristics of patients undergoing single-port and multi-port laparoscopic left lateral sectionectomy

Variable	SPLS (*n* = 42)	MPLS (*n* = 35)	*p*-value
Patient Demographics			
Age (years), median [IQR]	63[45–68]	65[52–67]	0.724
Sex (Male), n (%)	24 (57.1)	19 (54.3)	0.652
BMI (kg/m²), median [IQR]	25.6 [19.8–32.4]	27.2 [21.4–33.2]	0.348
**Liver Background**			
Child-Pugh Class A, n (%)	42 (100)	35 (100)	0.985
Cirrhosis(Metavir score ≥ F3), n (%)	14 (33.3)	12 (34.200)	0.732
**Pathological diagnosis**,** n (%)**			
**Malignancy**	**17 (40.5)**	**15 (42.9)**	**0.556**
Hepatocellular carcinoma (HCC)	12	10	
Colorectal liver metastasis (CRLM)	5	4	
Other metastatic tumors	0	1	
**Benign Disease**	**25 (59.5)**	**20 (57.1)**	**0.708**
Hemangioma	4	3	
Hepatolithiasis	17	11	
Benign cyst / Abscess	3	5	
Others	1	1	
**Tumor Characteristics**			
Tumor size (cm), median [IQR]	2.5 (0.9–5.3)	2.2(1.2-5.0)	0.782
Multiple tumors, n (%)	5 (11.9)	4 (11.4)	0.846

*Abbreviations*: *SPLS* single-port laparoscopic surgery; *MPLS* multi-port laparoscopic surgery; *ns* not significant

This table details the demographic and clinical characteristics of patients undergoing SPLS and MPLS. Continous variables are presented as median with interquartile range (IQR), and categorical variables as number (%). No significant differences were observed between the two groups

Regarding pathological diagnosis, malignant lesions were identified in 40.5% of SPLS and 42.9% of MPLS cases (*p* = 0.556), with hepatocellular carcinoma accounting for the majority of malignancies (12 vs. 10 cases). Benign lesions were predominant in both groups (59.5% vs. 57.1%, *p* = 0.708), most commonly hepatolithiasis. Tumor characteristics were similar, including median tumor size (2.5 vs. 2.2 cm, *p* = 0.782), and the proportion of multiple tumors (11.9% vs. 11.4%, *p* = 0.846).

### Intraoperative outcomes

All 77 procedures were completed laparoscopically without conversion to open surgery. In the SPLS group, all cases were successfully completed through a single umbilical incision without the requirement for additional trocars or conversion to multi-port surgery. Operative outcomes were comparable between the two groups. The median operative time was 145 min for SPLS and 130 min for MPLS (*p* = 0.340). Median estimated blood loss was 200 mL and 250 mL, respectively (*p* = 0.400). No patient in the SPLS group required an intraoperative transfusion, whereas two patients (5.7%) in the MPLS group received blood transfusions (*p* = 0.240). The R0 resection rate was 100% in both groups.

### Postoperative outcomes

Postoperative outcomes are presented in Table 2. The overall complication rate was low and not significantly different between groups (SPLS 2.4% vs. MPLS 5.7%; *p* = 0.60). In the SPLS group, one patient (2.4%) experienced a Clavien-Dindo grade IIIa subcapsular hematoma, successfully managed by percutaneous drainage. In the MPLS group, two patients (5.7%) developed complications: one grade II intra-abdominal fluid collection and one grade IIIa postoperative bleeding requiring angiographic embolization. There were no 30-day mortalities, reoperations, or port-site complications in either group. Notably, the SPLS group had a significantly shorter postoperative hospital stay compared with the MPLS group (median 5 days vs. 6 days; *p* = 0.020). No readmissions occurred in the SPLS group, while one patient (2.9%) in the MPLS group was readmitted for a non-surgical issue. Detailed postoperative complications are summarized in Table 3.

**Table 2:** Comparison of intraoperative and postoperative outcomes between single-port and multi-port laparoscopic left lateral sectionectomy

Variable	SPLS (*n* = 42)	MPLS (*n* = 35)	*p*-value
Intraoperative Outcomes			
Operative time (min), median [IQR]	145 [90–220]	130 [80–210]	0.341
Blood loss (mL), median [IQR]	200 [50–500]	250 [50–600]	0.406
Intraop transfusion, n (%)	0 (0)	2 (5.7)	0.248
R0 resection rate, n (%)	42 (100)	35 (100)	0.989
**Postoperative Outcomes**			
Postop complications, n (%)	1 (2.4)	2 (5.7)	0.602
Clavien-Dindo grade ≥ III	1 (2.4)	1 (2.9)	0.996
Hospital stay (days), median [IQR]	5 [3–8]	6 [4–10]	**0.020**
Readmission (30 days), n (%)	0 (0)	1 (2.9)	0.455

*Abbreviations*: *SPLS* single-port laparoscopic surgery; *MPLS* multi-port laparoscopic surgery; *ns* not significant.

Operative variables include operative time, estimated blood loss, transfusion requirement, and R0 resection rate. Postoperative outcomes include complication rate, Clavien-Dindo grade ≥ III complications, length of hospital stay, and readmission within 30 days

**Table 3:** Detailed postoperative complications

Complication	SPLS (*n* = 42)	MPLS (*n* = 35)	Clavien-Dindo Grade	Management
**Subcapsular Hematoma**	1 (2.4%)	0	IIIa	Percutaneous drainage
**Intra-abdominal Collection**	0	1 (2.9%)	II	Antibiotics and observation
**Postoperative Bleeding**	0	1 (2.9%)	IIIa	Angiographic embolization
**Total patients with complications**,** n (%)**		1 (2.4%)		2 (5.7%)

Abbreviations: *SPLS* single-port laparoscopic surgery; *MPLS* multi-port laparoscopic surgery. Note: Data are presented as *n* (%)

Postoperative complications are classified according to the Clavien-Dindo grading system, with corresponding management strategies described for each event

Overall, SPLS using articulating instruments achieved perioperative outcomes comparable to those of conventional MPLS, with the exception of a significantly shorter postoperative hospital stay. Importantly, all SPLS procedures were completed without additional port placement or conversion, supporting the technical feasibility of this approach in selected patients.

## Discussion

In this comparative study, we demonstrated that SPLS using articulating instruments can be performed as safely and effectively as conventional MPLS for left lateral sectionectomy in selected patients. Despite well-recognized ergonomic concerns associated with SPLS, key perioperative outcomes—including operative time, estimated blood loss, need for transfusion, R0 resection rate, and postoperative complication rates—showed no significant differences between the two groups [11–13]. Notably, SPLS was associated with a shorter postoperative hospital stay, suggesting potential benefits in recovery without compromising surgical safety.

A major strength of this study is the strict baseline comparability between the groups. Patient demographics, liver function, prevalence of cirrhosis, and tumor characteristics— including malignancy rate, size, and multiplicity—were evenly distributed. As detailed, patients were similarly distributed in terms of age, sex, BMI, liver function (all Child-Pugh A), prevalence of liver cirrhosis (33.3% vs. 34.3%), and tumor characteristics including malignancy rate (40.5% vs. 42.9%), median tumor size (2.5 vs. 2.2 cm), and multiplicity (11.9% vs. 11.4%). These findings support the interpretation that perioperative outcomes were not influenced by tumor biology or hepatic background but rather reflected the surgical approach itself.

Our results are consistent with previous reports demonstrating the feasibility of SPLS for minor liver resection [14–17]. Prior comparative studies and meta-analyses have reported similar operative time and blood loss between SPLS and MPLS, with a tendency toward shorter hospital stays in SPLS group [18, 19]. Interestingly, although transfusion events in our study occurred only in the MPLS group, both cases involved patients with underlying cirrhosis. This observation suggests that intraoperative bleeding risk may be more closely related to liver parenchymal quality than to the surgical port configuration, underscoring the importance of careful patient selection when considering SPLS.

A distinctive feature of our approach was the use of articulating laparoscopic instruments, which effectively address the inherent ergonomic limitations of single-port laparoscopic surgery. SPLS is constrained by parallel instrument insertion through a single access point, leading to instrument crowding and external and internal collision commonly described as the “sword fighting” or “chopstick effect” [20, 21]. These mechanical constraints limit effective traction-contertraction and compromise precise parenchymal manipulation. In contrast, articulating instruments enter in a crossed configuration but restore internal triangulation through wrist-like articulation. This design allows separation of working angles within the operative field despite a single-port entry, enabling stable traction, controlled dissection, and precise stapler application without instrument collision. The absence of additional trocar placement or conversion to open surgery in all SPLS cases suggest that articulating technology may mitigate technical barriers and potentially attenuate the learning curve traditionally associated with SPLS.

From a patient perspective, SPLS offers improved cosmetic outcomes by limiting the incision to a concealed umbilical site [22, 23]. Although postoperative pain scores and cosmetic satisfaction were not formally assessed in this study, previous reports consistently indicate higher cosmetic satisfaction following single-port procedures. The shorter hospital stay observed in the SPLS group may suggest reduced surgical trauma from the absence of additional trocar sites; however, differences in discharge criteria under ERAS protocols should also be considered [24, 25].

Despite these favorable findings, SPLS remains a technically demanding procedure [26]. The outcomes reported in this series likely reflect the experience of a single hepatobiliary surgeon with substantial prior expertise in laparoscopic liver surgery. While the single-surgeon design enhances internal consistency, it limits generalizability, particularly for surgeons early in their SPLS learning curve or centers without access to articulating instruments. Furthermore, the feasibility of a transumbilical single-port approach may be reduced in morbidly obese patients due to increased abdominal wall thickness, in whom alternative port strategies or robotic platform may be more appropriate.

When benchmarked against established “textbook criteria” for laparoscopic LLS—including major complication ≤ 5%, length of stay ≤ 6 days, mortality ≤ 1%—the outcomes of SPLS cohort in this study meet accepted international standards. In this context, the use of articulating instruments appears to facilitate safe implementation of SPLS by mitigating technical constraints inherent to single-port surgery, without compromising perioperative outcomes [27, 28]. More broadly, articulating devices may serve as a cost-efficient alternative to robotic systems, offering enhanced dexterity without requiring substantial capital investment (Table 4).

**Table 4:** Comparison of Single-Port LLS Outcomes with Established ‘Textbook Outcome’ Benchmarks

Performance Indicator	Textbook Outcome’ Benchmark	Current Study (SPLS, *n* = 42)	Comparison
**Mortality**	≤ 1%	0%	Met
**Major Complications (Clavien-Dindo ≥ III)**	≤ 5%	2.4% (*n* = 1)	Met
**Overall Complications**	≤ 20%	2.4% (*n* = 1)	Exceeded
**Blood Transfusion**	0	0%	Met
**R0 Resection**	100%	100%	Met
**Postoperative Hospital Stay**	≤ 6 days	5 days	Met
**Readmission within 30 days**	Not specified in benchmark, but part of ideal outcome	0%	Met

Benchmark criteria include mortality, major complications(Clavien-Dindo grade ≥ III), overall complications, blood transfusion, R0 resection rate, postoperative hospital stay, and 30-day readmission

## Conclusions

SPLS assisted by articulating instruments appears to be a safe and feasible alternative to conventional multi-port laparoscopic surgery in selected patients. By improving triangulation and ergonomic control, articulating devices mitigate the inherent technical limitations of single-port surgery, enabling perioperative outcomes comparable to MPLS. In this study, SPLS achieved operative safety, oncologic adequacy, and postoperative recovery profiles that met established international “textbook outcome” benchmarks for laparoscopic LLS, with the additional benefit of a shorter postoperative hospital stay. These findings suggest that, in experienced hands, articulating instrument-assisted SPLS can be performed without compromising surgical quality. However, the applicability of these results should be interpreted with caution. This study reflects the experience of a single surgeon at a single institution and included a carefully selected patient population with preserved liver function and relatively favorable body habitus. Therefore, the generalizability of SPLS—particularly in patients with obesity, advanced cirrhosis, or in centers early in the learning curve—remains to be established.

Future multicenter prospective studies with large cohorts, longer follow-up, and standardized assessment of patient-related outcomes are warrented to validate these findings, evaluate long-term oncologic and incisional outcomes, and more precisely define the role of articulating instrument-assisted SPLS within the spectrum of minimally invasive liver surgery.

## Data Availability

No datasets were generated or analysed during the current study.
